# Reproductive output of old males is limited by seminal fluid, not sperm number

**DOI:** 10.1093/evlett/qrae071

**Published:** 2025-01-06

**Authors:** Krish Sanghvi, Sucheta Shandilya, Alana Brown, Biliana Todorova, Martin Jahn, Samuel J L Gascoigne, Tara-Lyn Camilleri, Tommaso Pizzari, Irem Sepil

**Affiliations:** Department of Biology, University of Oxford, Oxford, United Kingdom; Department of Biology, University of Oxford, Oxford, United Kingdom; Department of Biology, University of Oxford, Oxford, United Kingdom; Department of Biology, University of Oxford, Oxford, United Kingdom; Department of Biology, University of Oxford, Oxford, United Kingdom; Department of Biology, University of Oxford, Oxford, United Kingdom; Department of Biology, University of Oxford, Oxford, United Kingdom; Department of Biology, University of Oxford, Oxford, United Kingdom; Department of Biology, University of Oxford, Oxford, United Kingdom

**Keywords:** aging, cryptic female choice, *Drosophila*, ejaculate, senescence, sexual selection

## Abstract

Male reproductive senescence is typically characterized by a decline in the number of sperm produced and transferred by old males, a phenomenon that may be exacerbated in polygynous species where males mate multiply. However, males also transfer seminal fluid to females, and little is known about its role in modulating male reproductive senescence. Here, we explore the contributions of sperm and seminal fluid towards male reproductive senescence in a series of sequential matings, using *Drosophila melanogaster*. As expected, old males produce fewer offspring than young males. However, this pattern is not driven by sperm limitation: old males have more sperm and transfer similar numbers to females, compared to young males. Instead, females storing fewer sperm of old males compared to that of young males, over a long term, drives male reproductive senescence. We are able to mitigate the age-related decline in male reproductive output by supplementing females with the seminal fluid of a young male, before she mates with an old male. Similarly, we alleviate the reduction in reproductive output across sequential matings by supplementing females with seminal fluid. Our findings highlight that seminal fluid, rather than sperm number, limits reproductive success in old or multiply mating males, highlighting its underappreciated role in reproductive aging.

## Teaser

What causes an old male to reproductively fail? Is it his sperm or seminal fluid? In fruit flies, old males produce fewer offspring than young males. But surprisingly, old males are not sperm limited. Instead, the reproductive decline is caused by females storing fewer sperm of old males. But why do females store fewer sperm of old males? Possibly because old males lack high-quality seminal fluid, which we demonstrate by rescuing the fertility of old males after supplementing the female with a young male’s seminal fluid.

## Introduction

Advancing age, often (Lemaître & Gaillard, 2017; Monaghan & Metcalfe, 2019), but not always (Jones et al., 2014; Sanghvi et al., 2024b), leads to irreversible declines in fecundity and fertility (Vrtílek et al., 2023), a pattern known as reproductive senescence (Monaghan et al., 2020; Pizzari et al., 2008; Vrtílek et al., 2022). In males, reproductive senescence is primarily attributed to age-dependent deterioration in mating success likely underpinned by senescence in precopulatory traits (Amin et al., 2012; Rezaei et al., 2015), or sperm quality (Dean et al., 2010; Gasparini et al., 2019; Johnson et al., 2015; Vuarin et al., 2019), quantity (Cornwallis et al., 2014; Sasson et al., 2012), and performance (Aich et al., 2021; Gasparini et al., 2010). However, male reproductive senescence can also occur due to deterioration in the non-sperm component of the ejaculate, seminal fluid (SF) (Fricke et al., 2023).

Understanding male reproductive senescence requires an integrated study of both, sperm- and SF-mediated changes in male reproductive output with age (Fricke & Koppik, 2019; Koppik & Fricke, 2017), because each can senesce at a different rate or have distinct ages at which senescence onsets (Reinhardt et al., 2011; Sepil et al., 2020). In many species, SF is crucial for ensuring fertilization (Chapman & Davies, 2004), maintaining sperm performance (den Boer et al., 2010; Ramm, 2020), promoting female oviposition (Heifetz et al., 2005; Poiani, 2006; Sirot et al., 2009b), and facilitating long-term sperm storage (Avila et al., 2011; Hopkins et al., 2017). SF of old males often has an altered SF protein (SFP) composition (Koppik & Fricke, 2017; Sepil et al., 2020), is of a lower quantity (Ruhmann et al., 2018) with more degraded SFPs (Fricke et al., 2023), or has higher levels of oxidative stress (Noguera et al., 2012) than SF of young males. Thus, age-related changes in SF can play an important role in constraining the reproductive output of old males.

Male reproductive senescence might be particularly relevant in polygamous species (Radwan, 2003), for example, where males can mate with multiple females in quick succession. Ejaculate production is costly (Dowling & Simmons, 2012; Olsson et al., 1997), and males in polygynous species can become sperm (Gerofotis et al., 2015; Pitnick & Markow, 1994; Rubolini et al., 2007) and SF depleted (Hopkins et al., 2019b; Linklater et al., 2007; Reinhardt et al., 2011; Sirot et al., 2009a) when mating multiply. Such ejaculate limitation can decrease the reproductive output of polygynous males as they progress through series of successive matings (Abe, 2019; Lewis, 2004; Loyau et al., 2012; Macartney et al., 2020; Swierk et al., 2015). In such scenarios, the effects of male reproductive senescence might become more pronounced later in a mating sequence if old males suffer steeper rates of ejaculate depletion (Bressac et al., 2008, 2009). For instance, terminal investment could lead old males to invest relatively more in early matings of a mating sequence, at the cost of lower investment in later matings (Duffield et al., 2017; Part et al., 1992), causing a steeper decline in reproductive output for old males through a mating sequence. Test of such hypotheses (see Supplementary Appendix 1 for additional hypotheses), however, are surprisingly lacking.

We use the fruit fly, *Drosophila melanogaster*, to investigate changes in the reproductive output of old and young males over a mating sequence, and subsequently investigate the ejaculate-mediated mechanisms underpinning these patterns. Previous studies in fruit flies show that old males have lower quality and quantity of sperm (Sepil et al., 2020; Turnell & Reinhardt, 2020) and SF (reviewed in Fricke et al., 2023), and lower reproductive success (Sanghvi et al., 2024a; Snoke & Promislow, 2003) than young males. Male fruit flies are polygynous (Hopkins et al., 2019b) and, due to a low sperm-to-egg number ratio (Bjork & Pitnick, 2006), can become ejaculate depleted after few matings (Hopkins et al., 2019b; Pitnick & Markow, 1994). We first test if advancing age leads to senescence in reproductive output of males in a mating sequence (Experiment A and Experiment B). In Experiment B, we further test if senescence in reproductive output is due to sperm limitation as is widely assumed. Finally, we investigate whether senescence in SF, the somatic component of the ejaculate, might explain declines in male reproductive output with age (Experiment C).

## Methods

### Stock maintenance

All flies in our experiments were maintained at a 12:12 hr light cycle at a constant temperature of 25 °C and 45% relative humidity, under which, flies have an egg-to-adult developmental time of 10 days. We used males of the wild-type Dahomey strain (“*dah*”) in Experiment A. In Experiment B, we additionally used *Ub-GFP* males (“*gfp*”) where transgenes expressing green fluorescent proteins enable the visualization of sperm heads (Manier et al., 2010). In Experiment C, we also used *son-of-tudor* (henceforth, “*sot*”) males which are infertile (i.e., sperm-less) but transfer SF (Boswell & Mahowald, 1985; Hopkins et al., 2019a; Kalb et al., 1993; Sepil et al., 2020; see Supplementary Appendix 2). All females used in our experiments were young (3–4 days old) and of *dah* background. Across all experiments, male flies between 3 and 11 days of age were considered young, and between 37 and 46 days as old, based on previous studies (Aguilar et al., 2023; Ruhmann et al., 2018; Sanghvi et al., 2024a; Sepil et al., 2020; Snoke & Promislow, 2003; Turnell & Reinhardt, 2020).

### Experimental flies

To generate experimental males of each line (*dah*, *gfp*, and *sot*), we collected eggs from our stock populations with a standardized egg density of ~150 flies per bottle (Clancy & Kennington, 2001). All experimental males and females were fed with Lewis medium supplemented with molasses and ad libitum live yeast (Lewis, 1960). All experimental flies were kept as virgins in single-sex vials of 10 individuals, until being used for the experimental assays. Experimental virgin males were transferred onto new food once a week. Each experiment was conducted across two to three cohorts (blocks), i.e., subpopulations of males from the same strain assayed on different days within an experiment, to reduce experimental noise. Sample sizes across the three experiments and survival curves of stock males in the three fly lines are provided in Supplementary Figure S1 and Supplementary Table S1, respectively. *Gfp* males were sampled at younger ages, yet experienced higher rates of mortality compared to *sot* and *dah* males. Such discrepancy between the biological and chronological ages of males in different strains might have made our three experiments less comparable with each other.

### Mating experiments

#### Experiment A

We first compared changes in the reproductive output of young (9–10 days old) and old (44–46 days old) males in a multiple-mating sequence. On the day of the mating assay, 60 old and 60 young experimental *dah* males were haphazardly chosen to successively mate with a maximum of *dah* 10 females, over a duration of 9 hr. Each male was moved into a vial containing a single young virgin female and pairs were directly observed by continuously scanning vials. Each male remained with a female until the pair mated, and if a male did not mate with a female by the end of the 9 hr assay, that female was not included in our analysis. Once mated, the male was immediately transferred into another vial containing a new, young virgin female. Mated females were given 24 hr to oviposit in the same vial, after which females were discarded. After 14 days of development, these vials were frozen at −20 °C, and the number of eclosed (adult) offspring counted.

#### Experiment B

Next, we investigated whether sperm limitation might drive differences in reproductive output of old and young mate-multiplying males. Specifically, we compared the number of sperm transferred to, and stored by, females mated to old or young mate-multiplying males, as well as the sperm reserves and accessory gland (AG) size of mate-multiplying old or young males. For this, we first generated experimental old (37–41 days old) and young (3–7 days old) *gfp* males. We then haphazardly chose 50 old and 28 young males to successively mate with a maximum of 10 young virgin *dah* females, over a duration of 9 hr. Old males are less likely to mate thus more were used. Like Experiment A, each male was moved into a vial containing a single *dah* female, and following a mating, males were immediately transferred into a new vial containing a different female. Following mating, odd-ranked females in a male’s mating sequence (i.e., 1st, 3rd, 5th, 7th, and 9th female) were given 24 hr to oviposit in the same vial, after which the female was frozen at −20 °C. The vials were frozen 14 days after oviposition, and the number of eclosed (adult) offspring in these vials was counted. All even-ranked females in a male’s mating sequence (i.e., the 2nd, 4th, 6th, 8th, and 10th female) were frozen within 30 min of mating, at −20 °C. Additionally, all mated *gfp* males as well as four old and nine young virgin *gfp* males not exposed to females were frozen at −20 °C after the mating assay. Virgin males were frozen to obtain a baseline for how many sperm were present in males without the effects of mating.

Reproductive tracts of frozen females (bursa, seminal receptacle, and spermathecae) and males (seminal vesicle [SV] and AGs) were dissected in PBS and later imaged (Supplementary Appendices 3–5). Sperm counts and AG size measurements were done using the image processing software, FIJI (previously called Image J; Schindelin et al., 2012).

#### Experiment C

Finally, we tested whether senescence in SF might explain differences in the reproductive output of old and young multiply-mated males. To test this, we investigated whether SF obtained by a female from her first mating (with a young *sot* male) impacts the reproductive output of old or young *dah* males who subsequently mate with these females. For this, we generated young (4–11 days old) virgin *sot* males which lack sperm but produce SF (see Supplementary Appendix 2). We mated ~200 young (3–4 days old) virgin *dah* females, each with a young virgin *sot* male, and observed their matings. On the same day, we then conducted a multiple-mating assay using these *sot*-mated *dah* females, and experimental young (9–10 days old) and old (44–46 days old) *dah* males as previously described in Experiment A. Specifically, we chose 115 old and 85 young experimental *dah* males haphazardly to successively mate with up to 10 *sot*-mated *dah* females, over a duration of 9 hr. Each young or old experimental *dah* male was moved into a vial containing a single *sot*-mated *dah* female and observed. Once mated, experimental *dah* males were immediately transferred into a new vial containing a different, young *sot*-mated *dah* female. Females who mated with experimental *dah* males were given 24 hr to oviposit in the same vial, after which females were discarded. These vials were frozen 14 days after the oviposition period at −20 °C, and the number of eclosed (adult) offspring in the vials was later counted.

### Data analysis

#### Male reproductive output

##### Experiments A, B, and C

We analyzed data on male reproductive output through a mating sequence from each of the three experiments separately, using the package *glmmTMB* (Brooks et al., 2017) in R v4.2 (R Core Team, 2012). In each of these analyses, the number of offspring produced (i.e., reproductive output) by each female over 24 hr of oviposition was our dependent variable. We included male age (young or old), female rank (henceforth, female number) in a mating sequence (1–10), their two-way interaction (i.e., Male age × Female number), and block as fixed effects, with male ID as a random effect across all models. Additionally, we included observation level as a random effect to control for overdispersion in the data (Harrison, 2014), which we assessed using *DHARMa* (Hartig & Hartig, 2017). Each analysis involved two steps. First, we ascertained whether including a zero-inflation term improved model fit. We did this by comparing a model with Poisson against one with a zero-inflated Poisson error distribution and chose the model with the lowest Akaike information criterion (henceforth, best-fit distribution). Second, using our best-fit distribution model, we compared three different fixed-effect structures pertaining to whether changes in male reproductive output through a mating sequence was linear or not. These model comparisons were done using a likelihood ratio test with the function *anova* in the package *base* (R Core Team, 2012). These fixed-effect structures were: a model where male age interacted with only the linear term of female number (Age * Female number); one where male age interacted separately with both the linear and quadratic term for female number ((Age * Female number) + (Age * I(Female number^2))); and one with male age, a linear term for female number, their two-way interaction, and a separate quadratic term for female number ((Age * Female number) + I(Female number^2)). Males that did not copulate with any female were excluded from these analyses.

#### Sperm transfer and storage

We compared the number of sperm in the SVs of old and young males in Experiment B. Here, we included male age, male mating success (i.e., total number of females a male mated with), their two-way interaction, and block as fixed effects. We modeled sperm counts as our dependent variable (one value per male). Similar to the models on reproductive output described above, for our model on sperm numbers in males, we first determined the best-fit error distribution. We then determined the most suitable fixed-effect terms pertaining to male mating success and whether to include them as a linear or quadratic term.

We additionally analyzed data on the number of sperm transferred to even-ranked females, as well as the number of sperm stored by odd-ranked females after 24 hr of egg laying, in two separate models. To test how male age affected the number of sperm transferred by males, we modeled sperm numbers in even-ranked females’ reproductive tracts as our dependent variable. To test how male age influenced the numbers of sperm stored by females after 24 hr, we modeled sperm numbers in odd-ranked females’ reproductive tract as our dependent variable (see Supplementary Appendices 3 and 4). For both models on sperm number in females, we modeled male age, female number in a mating sequence, their two-way interaction, and block as fixed effects, with male ID and an observation level as random effects. Our model selection procedure for both models was the same as described above and involved determining the best-fit error distribution, and then determining whether female number was to be modeled as linear or quadratic.

Lastly, we analyzed data on the area of AGs (which are the primary site of SF production) on a subset of males. For this, we created a linear model with Gaussian error distribution in the *lme4* (Bates et al., 2014) package and included male AG area (one value per male, which was the average area of the two AGs) as our dependent variable. Male age, male mating success (i.e., total number of females a male mated with), their two-way interaction, and block were modeled as fixed effects. All linear mixed models (LMMs) were checked for normality of residuals and homoscedasticity using the *stats* package (R Core Team, 2012).

#### General notes on analysis

For all our models, female number was included as an ordinal variable. Generally, only when the two-way interactions between male age and female number or male mating success were nonsignificant, we created a main-effects model to interpret the independent influence of fixed effects (Engqvist, 2005). We conducted post-hoc pairwise comparisons between old and young males using effect sizes (Hedge’s g, with α = 0.05) for each female number in a mating sequence. Final model structures and the variance explained by fixed effects in final models (*R*^2^_marginal_) are described in Supplementary Table S2.

## Results

### Experiment A

**Figure 1.**
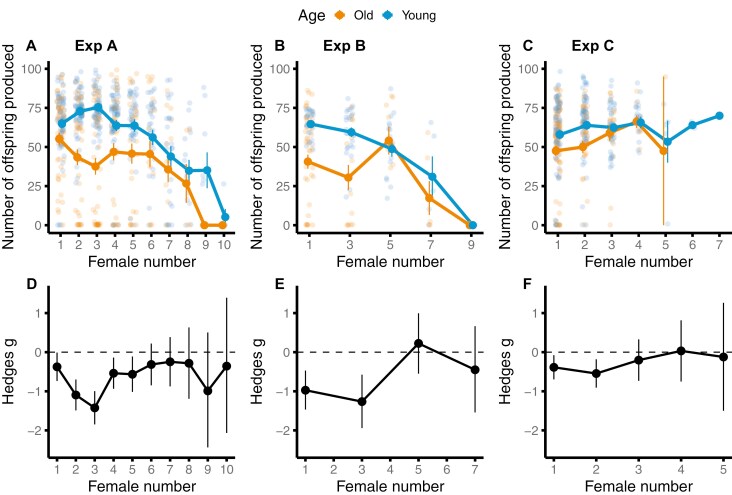
Reproductive output of old and young males in a mating sequence in Experiments A, B, and C. (A–C) Effect of male age and female number in a male’s mating sequence on the number of offspring produced by mated females, for Experiments (Exp) A, B, and C, respectively. Means and SEM are shown. (D–F) Post-hoc pair-wise comparisons between young and old males using effect sizes (Hedges’ *g*) of reproductive output with each female in a mating sequence, for Experiments A, B, and C, respectively. Means and 95% CIs are shown. When CI does not overlap with zero, the effect is significant for α = 0.05. Negative values indicate old males having lower reproductive output than young males.

We first compared offspring production between old and young *gfp* males through a multiple-mating sequence. Variation in the number of offspring produced by mated females was explained by a significant two-way interaction between male age and female number in a mating sequence (*z* = −2.24; *p* = 0.025; Figure 1A, Supplementary Table S3). Post-hoc tests (Figure 1D) revealed that young males produced significantly more offspring compared to old males, earlier in a mating sequence. Overall, male reproductive output declined through a mating sequence; however, this decline was steeper for young than old males (Supplementary Figure S2).

### Experiment B

Next, we compared offspring production and numbers of sperm retained by old or young *gfp* males, and sperm numbers transferred to and stored by females mated to these males in a mating sequence. Patterns of offspring production in Experiments A and B were similar. However, there was no significant interaction between male age and female number in the mating sequence, on the number of offspring produced (*z* = −1.14, *p* = 0.254, Figure 1B, Supplementary Table S4). Yet, old males produced fewer offspring than young males (*z* = 2.01, *p* = 0.044), and males produced fewer offspring with females later in a mating sequence (*z* = −3.23, *p* = 0.001; Figure 1B and E, Supplementary Figure S3, Supplementary Table S4).

**Figure 2.**
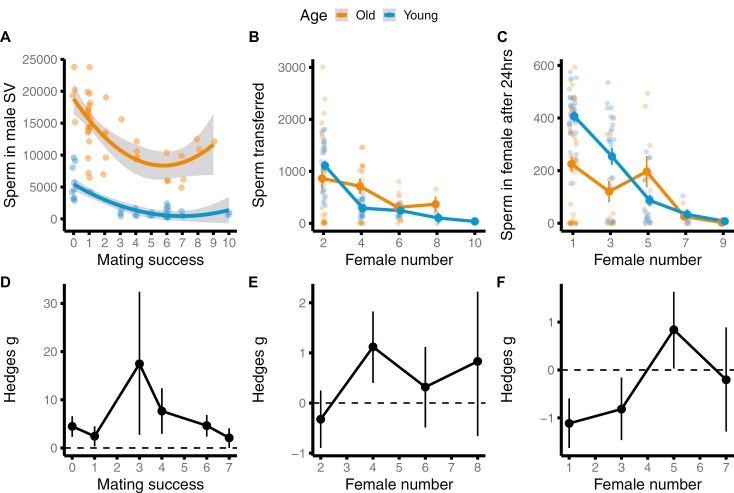
Age-dependent sperm accumulation and allocation through a mating sequence in Experiment B. (A) Number of sperm in male seminal vesicles (SVs) for old and young males with different mating success. (B) Sperm transferred to females by young and old males in a mating sequence. (C) Sperm stored by females mated to young and old males, after 24 hr. Means and SEM are shown for panels A–C. (D–F) Associated effect sizes when comparing young and old males in panels A–C, respectively, for each female number/mating success value. Means and CIs are shown for panels D–F. For panels D–F, when CI does not overlap with zero, the effect is significant for α = 0.05; negative values indicate that old males have lower sperm numbers than young males.

We then tested whether this pattern was driven by differences in sperm number reserves in old versus young males. We found a significant interaction between male age and mating success (i.e., total number of females a male mated with), on the number of sperm present in a male’s SV (*z* = −4.63, *p* < 0.001, Figure 2A, Supplementary Table S5). Old males had consistently more sperm present in their SV than young males, but this difference was greatest for males with intermediate mating success (Figure 2A and D). Hence, age-related differences in offspring number could not be explained by older males being sperm limited.

We then explored whether the lower reproductive output of old males could be attributed to old males transferring fewer sperm to females. We found a significant two-way interaction between male age and female number in a mating sequence, to affect the number of sperm transferred to females (*z* = −2.905, *p* = 0.004, Supplementary Table S6). Old and young males transferred similar numbers of sperm to females early in their mating sequence. However, old males tended to transfer more sperm to females later in the mating sequence compared to young males (Figure 2B and E, Supplementary Figure S4). Therefore, age-related differences in offspring number were unlikely to be explained by old males transferring fewer sperm to females, compared to young males.

Next, we tested whether the low reproductive output of old males could be due to females storing fewer sperm (24 hr after mating) when mated to old males, compared to young males. We found a significant two-way interaction between male age and female number in the sequence (*z* = −2.030, *p* = 0.042, Supplementary Table S7). Females mated to young males stored more sperm than females mated to old males, early in the mating sequence (Figure 2C and F, Supplementary Figure S5A). Visual inspection revealed an asymptotic relationship between the number of sperm stored by females after 24 hr, and the number of offspring produced by these females over 24 hr (Supplementary Figure S5B). Male age-related changes in sperm numbers stored by females were consistent with age-related changes in reproductive output of males, suggesting that the low reproductive output of old males is likely due to females storing fewer sperm of old males, over a long-term.

**Figure 3.**
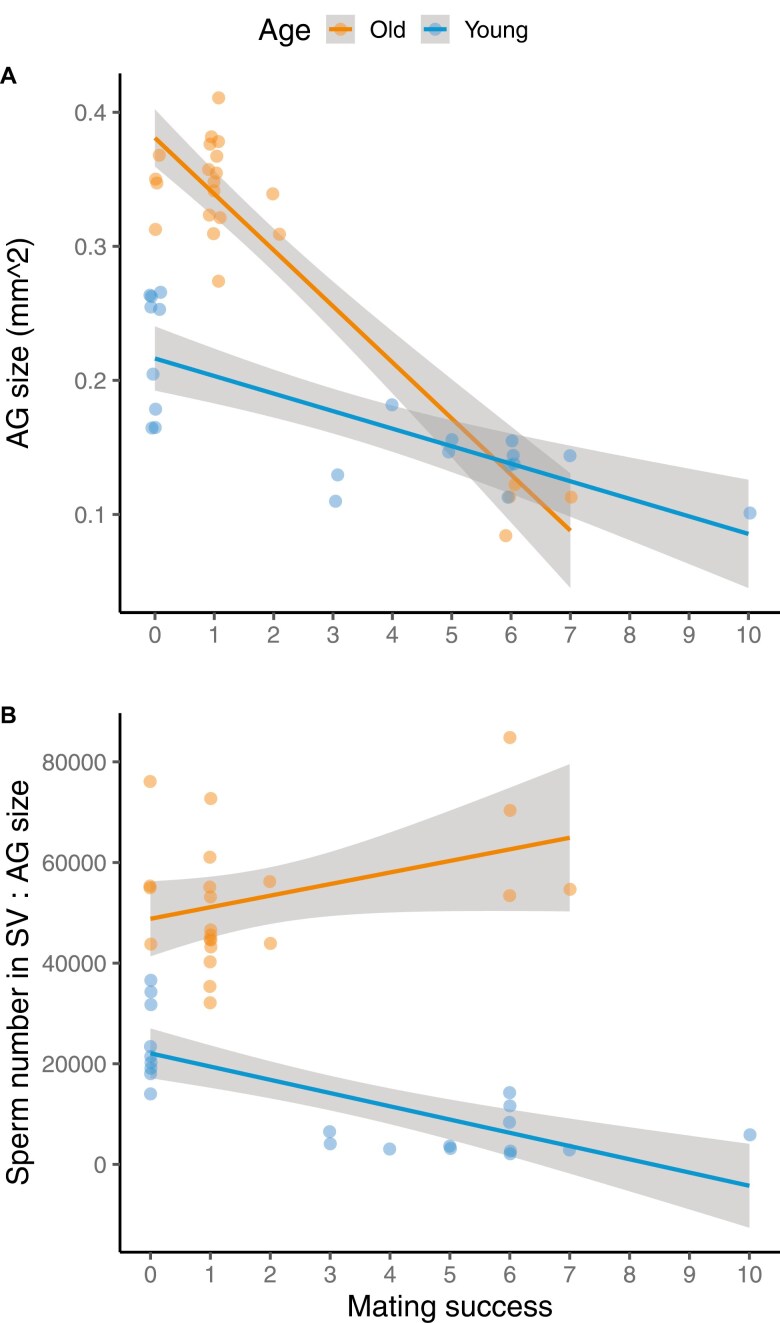
Relative and absolute changes in accessory gland (AG) size in old and young *gfp* males of different mating success in Experiment B. (A) Significant effect of the interaction between male age and male mating success, to affect the size of male AGs (mm^2^). Old males, despite having larger AGs when virgins, show steeper declines in AG size than young males through a mating sequence. Means and 95% CIs are shown. (B) Relative abundance of sperm versus seminal fluid (AG size used as proxy) in old and young males as a function of mating success. When virgin, old males exhibit a higher sperm-to-AG size ratio compared to young males, suggesting that seminal fluid is relatively more limiting for older males than sperm. This disparity becomes more pronounced with increasing mating success, indicating an even greater relative limitation of seminal fluid in older males compared to younger ones. Young males were 3–7 days old, and old males were 37–42 days old.

In Experiment B, we also indirectly tested whether accumulation and allocation of SF might explain the reproductive output differences between old and young males seen in Experiments A and B. We found a significant interaction between male age and mating success (*z* = 6.577, *p* < 0.001, β_old_ = −0.042, β_young_ = −0.013, Supplementary Table S8) on AG size. Old males had larger AG than young males when virgins; however, the rate of decline in AG size was steeper for old than young males (Figure 3A and B). Therefore, later in a mating sequence, AG size did not differ between old and young males. These results suggest that old males might become SF depleted at a faster rate through a mating sequence than young males.

### Experiment C

Finally, we tested if the decline in male reproductive output is driven due to females being SF limited, by providing “extra” SF to females via mating them with *sot* males. When females were supplemented with the SF of a young male, there were no significant effects of male age (*z* = 1.65, *p* = 0.099), female number in a mating sequence (*z* = 1.3, *p* = 0.193), or their interaction, on the number of offspring produced (*z* = −1.46, *p* = 0.145, Figure 1C and F, Supplementary Figure S6; Supplementary Table S9). These results suggest that SF from young males might be sufficient to rescue male reproductive senescence.

In all three experiments, old males mated with fewer females (i.e., had lower mating success) compared to young males, which could be associated with longer mating latencies of old males (Supplementary Appendix 6, Supplementary Figure S7).

## Discussion

### Summary of findings

We investigated age-related differences in male reproductive output across a mating sequence and tested whether these patterns were driven by limitations in sperm or SF. Old males generally had lower reproductive output than young males. Surprisingly, however, old males had larger sperm reserves and transferred similar or more sperm to females, but females mated to old males stored fewer sperm of old males. Supplementing females with SF from young males reduced differences in reproductive output between old and young males and mitigated fertility declines across the mating sequence. Despite having larger AGs, old males experienced faster reduction in AG size through the mating sequence.

### Changes with male age

We found that old males had higher numbers of sperm stored in their SVs than young males (also shown by Bressac et al., 2009; Decanini et al., 2013; Kehl et al., 2013, 2015; Sepil et al., 2020). Sanghvi et al. (2024b) in a meta-analysis found this pattern across insects and suggest that experimental males typically being maintained as virgin might lead to longer periods of sperm accumulation in old than young males. In our study, we maintained males as virgins until the mating assay, thus old males might have accumulated more sperm than young males because spermatogenesis occurs throughout adult life in fruit flies with relatively low levels of sperm loss (Bjork et al., 2007; Demarco et al., 2014; Santos et al., 2023; Sepil et al., 2020). Selective disappearance of males (Bouwhuis et al., 2009; Sanghvi et al., 2022) with low sperm production rates could have also led to old males having higher sperm numbers than young males.

Despite having larger sperm reserves and transferring more or similar numbers of sperm to females, females stored fewer sperm from old males (Experiment B), and old males generally produced fewer offspring than young males (Experiments A and B). Age-related changes in SFP composition or quality might explain these results. In Experiment C, the prior receipt of SF by females resulted in similar number of offspring produced by old and young males. In fruit flies, SFPs produced in AGs play an important role in fertilization, female sperm uptake and storage, and ovulation (Avila et al., 2011; Bloch Qazi & Wolfner, 2003; Chapman & Davies, 2004; Heifetz et al., 2005; Poiani, 2006; Ravi Ram & Wolfner, 2007). Old *D. melanogaster* males have lower expression of SFP genes such as ovulin, sex peptide, and Acp36DE and have more degraded SFPs compared to young males (Fricke et al., 2023). Thus, sperm of old *dah* males might have benefitted from being exposed to the SF of young *sot* males, alleviating senescence in old males. A recent study shows that in females, exposing old oocytes to young follicular fluid alleviates senescence in oocytes (Wang et al., 2024). Similar techniques could be explored for rescuing senescence in old sperm, by exposing it to young SF.

Our finding of old males transferring similar sperm numbers as young males, but producing fewer offspring, might also be explained by variation in *quantities* of SF transferred. We found that old males had larger AGs than younger males (Reinhardt et al., 2011; Sepil et al., 2020). However, AG size may not always be a good proxy for SF quantity (e.g., Santhosh & Krishna, 2013), if for instance, old males have larger AGs due to underlying pathologies (Fricke et al., 2023). Future studies could manipulate the age of *sot* (SF-donor) males, as well as measure the quantity of SF transferred to females, to disentangle whether quality, composition, or quantity of SF explains our results better. Our results have important implications for sexual selection and conflict because they demonstrate that males who mate second might indirectly benefit from the SF of males who mate first with a female (Alonzo & Pizzari, 2010; Holman, 2009; Nguyen & Moehring, 2018), in addition to benefiting from last male sperm precedence. Future studies could compare senescence rates of SF and the somatic tissues producing it, against senescence in the germline and sperm, to test the disposable soma hypothesis at the ejaculate level, i.e., whether somatic tissues senesce at faster rates than the germline (Maklakov & Immler, 2016).

Male age-related changes in sperm viability and cryptic female choice might have also contributed to the results we found. Old males might have less viable or motile sperm than young males (e.g., Sturup et al., 2013), leading to fewer sperm to be stored by females mated to old males despite transferring similar or more sperm numbers compared to young males, consequently resulting in reduced fertilization rates for old males. Alternatively, females mated to old males might have ejected greater proportions of sperm than females mated to young males, via cryptic female choice (Snook & Hosken, 2004; Vuarin et al., 2019; Wagner et al., 2004). We found young males to have shorter mating latencies than old males, early in a mating sequence, suggesting a precopulatory preference of females towards young males. It is possible that such preference for young males might also occur postcopulation via cryptic female choice (Eberhard, 2015).

### Changes through a mating sequence

We found that males produced fewer offspring as they progressed through a mating sequence in Experiments A and B. Studies have previously shown that males, when mating multiply, can become depleted of sperm and SF (Gerofotis et al., 2015; Pitnick & Markow, 1994; Rubolini et al., 2007), producing fewer offspring with females encountered later in a mating sequence (Abe, 2019; Lewis, 2004; Loyau et al., 2012; Macartney et al., 2020; Swierk et al., 2015). The asymptotic relationship between sperm stored in females and offspring produced by females in our study indicates that reproductive output through a mating sequence is likely limited by SF, not sperm numbers (Hopkins et al., 2019b; Sirot et al., 2009a). Here, even if males transferred more sperm, offspring production would not increase because female ovulation and fertilization rates are constrained by insufficient quantities of SF (Bloch Qazi et al., 2003; Chapman, 2001). Results from Experiment C support this. Here, females were provided with SF from a previous mating before the focal mating took place, leading to focal-male reproductive output not declining through the mating sequence. Fruit fly males become depleted of SF at faster rates than sperm, when mating multiply (Linklater et al., 2007; Reinhardt et al., 2011), which is consistent with results of our study.

A lack of decline in reproductive output through the mating sequence in Experiment C might also be explained by male ejaculate plasticity. Females in this experiment were first mated to *sot* males before being used to mate with focal *dah* males. Male flies can detect female mating status using olfactory cues (Friberg, 2006; Lupold et al., 2011) to infer levels of sperm competition. Cues of high sperm competition risk (i.e., many rival males) lead male flies to transfer larger ejaculates to females (Bretman et al., 2009; Hopkins et al., 2019b; Wedell & Cook, 1999) and even alter their SFP composition (Ramm et al., 2015; Wigby et al., 2009). Hence, in Experiment C, focal *dah* males could have perceived higher sperm competition levels and increased their ejaculate investment in each mating, reducing reproductive decline through the mating sequence, possibly at the cost of fewer overall matings in Experiment C compared to Experiments A and B. This ejaculate plasticity could have confounded the rescuing effects of SF, which future studies could disentangle.

## Conclusions

Male reproductive senescence is multifaceted and can be exacerbated under polygyny, due to differential patterns of ejaculate accumulation and allocation between old and young mate-multiplying males. We show that different components of the ejaculate—sperm versus SF—decline at different rates with age and through a mating sequence. Old males have lower reproductive output than young males, which is likely driven by senescence in SF, sperm viability, or differential sperm storage by females, rather than senescence in sperm number. These results have important consequences for sexual conflict and sexual selection (Adler & Bonduriansky, 2014; Damiens & Boivin, 2006; Carazo et al., 2011; see Supplementary Appendix 7), whereby female fitness is not only determined by a male’s mating history (e.g., Wedell et al., 2002) or male age (Monaghan & Metcalfe, 2019), but also their interaction. Importantly, our results show that declines in male reproductive output are not permanent and can be reversed by the presence of sufficient, high-quality SF. This interplay between sperm and SF has crucial implications for our understanding of aging and reproductive biology, as well as potential biomedical applications.

## Supplementary material

Supplementary material is available online at *Evolution Letters*.

qrae071_suppl_Supplementary_Material

## Data Availability

All data from our experiment and the R code used for analysis can be found at OSF: https://osf.io/5z7m3/ Data are also available at Dryad at https://doi.org/10.5061/dryad.mpg4f4r99. Images of dissected *gfp* males’ SV, AGs, and images for sperm numbers transferred to and stored by females can be found on figshare: https://doi.org/10.6084/m9.figshare.25407223.v1, https://doi.org/10.6084/m9.figshare.25407253.v1, https://doi.org/10.6084/m9.figshare.25407382.v1.
