# Supplementary material for: Reproductive output of old males is limited by seminal fluid, not sperm number
Source: Evol Lett. 2025 Jan 6;9(2):282–91. doi: 10.1093/evlett/qrae071 (PMC11968187; doi:10.1093/evlett/qrae071)
Supplement: qrae071_suppl_Supplementary_Material [file qrae071_suppl_supplementary_material.docx]

**Supplementary material**

(Supplementary figures, Supplementary tables, Appendix)

Supplementary figures (S1-S7)


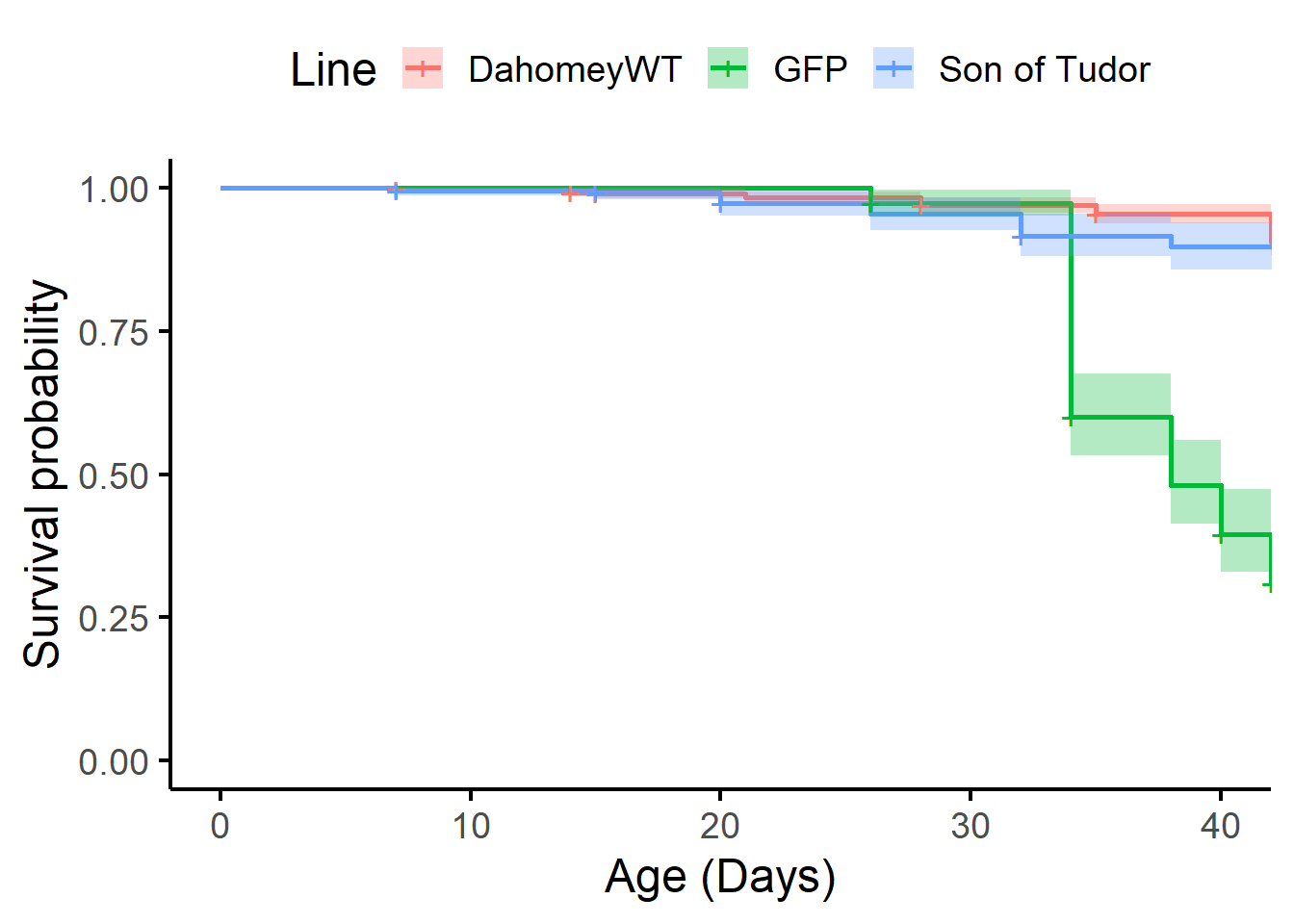


Figure S1: Age-dependent survival probability of males used in our study, from three distinct lines of *Drosophila melanogaster: dah, gfp, and sot.* *Gfp* males experienced higher mortality than *sot* or *dah* males. Means and 95% C.I. shown.


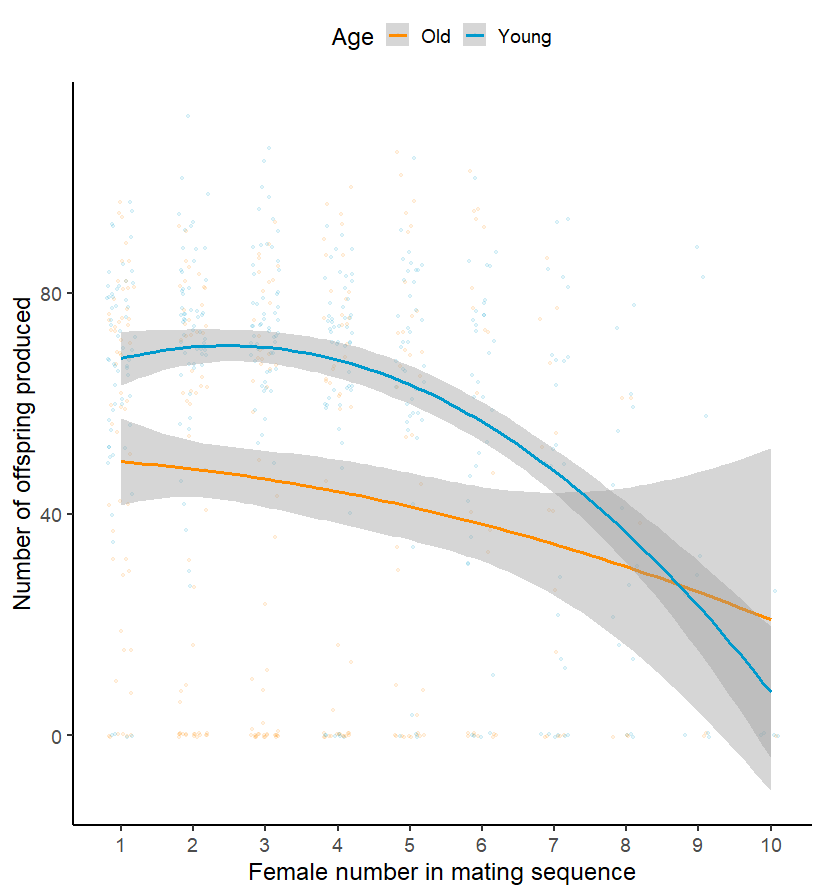


Fig S2: Significant interaction between male age and female number in a male’s mating sequence, to affect the number of offspring produced by a female over 24 hours of egg laying, in Experiment A. Old males produce fewer offspring than young males only early on in a mating sequence. Means and 95% C.I. shown.


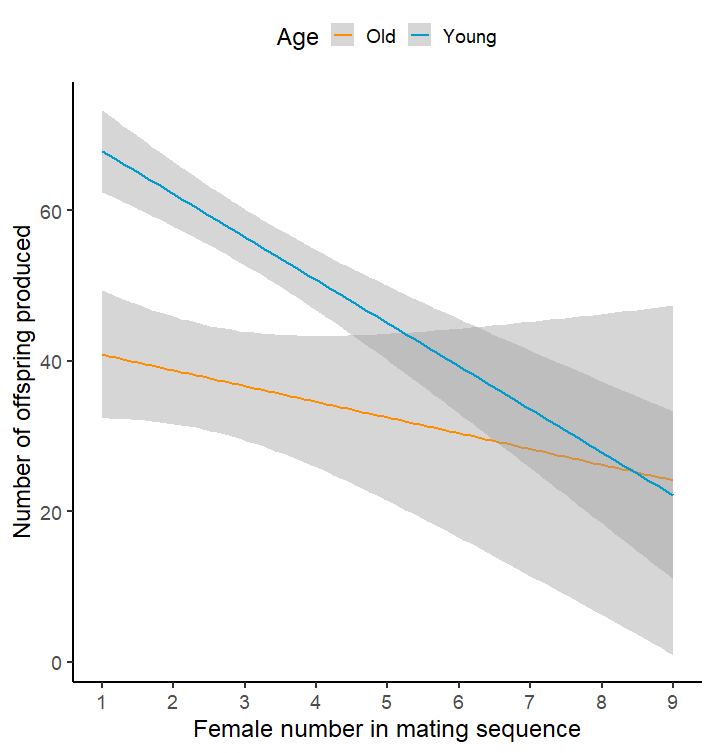


Fig S3: Significant effect of male age and female number in a male’s mating sequence, to affect the number of offspring produced by a female over 24 hours of egg laying, in Experiment B. Old males produce fewer offspring than young males, and males produce fewer offspring with females later than earlier in a mating sequence. Means and 95% C.I. shown.


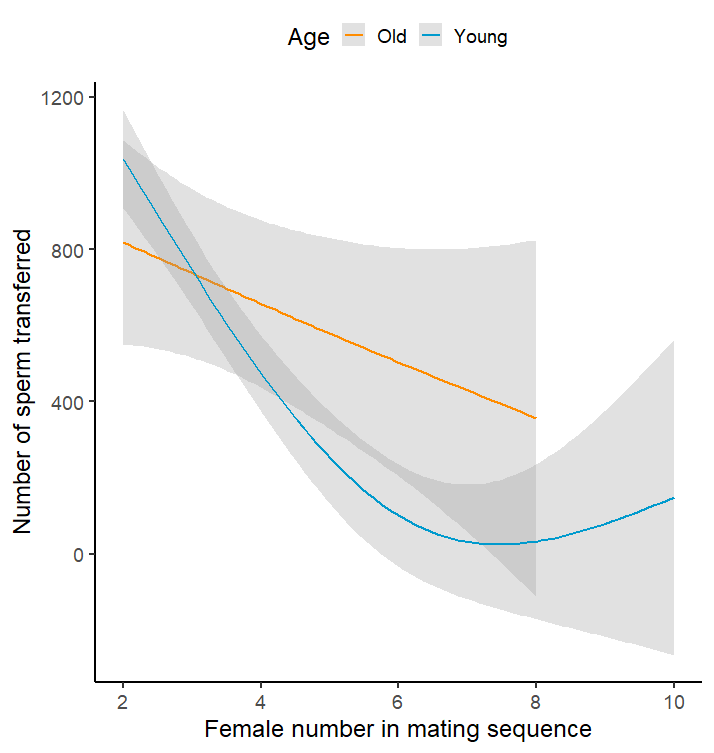


Figure S4: Effect of male age and female number in a male’s mating sequence, on the number of sperm transferred by the male to the female, in experiment B. Means and 95% C.I. shown.


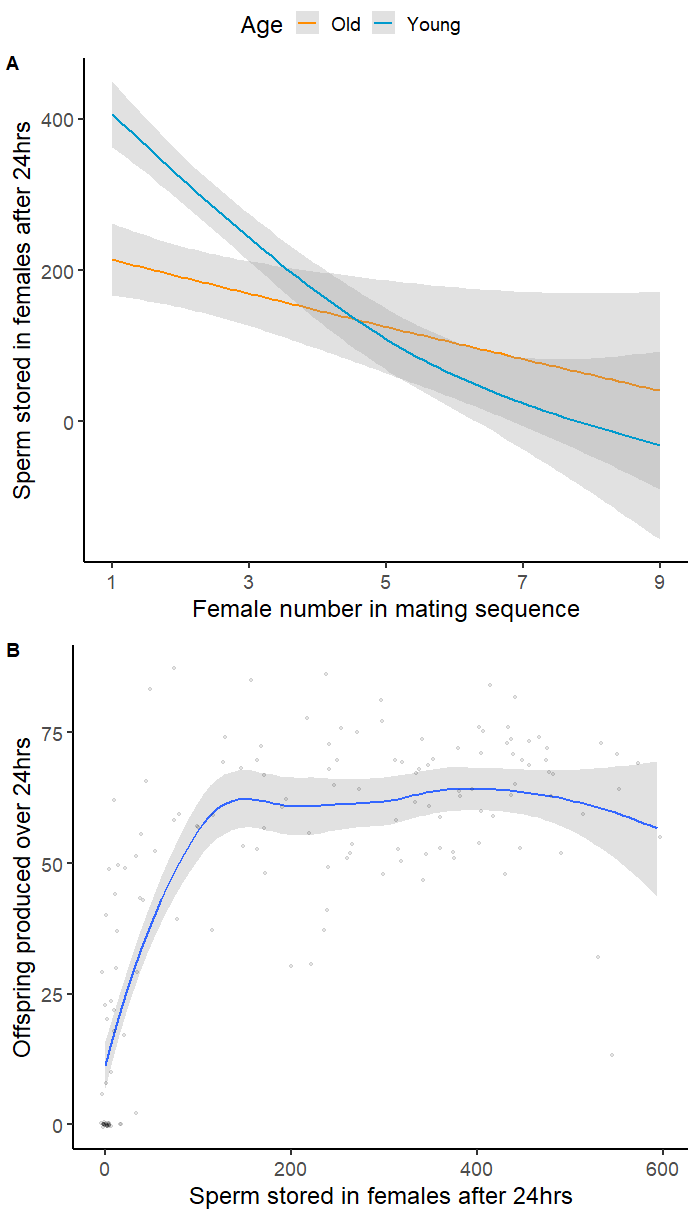


Figure S5: Experiment B. 5A- Effect of male age and female number in a male’s mating sequence, on the number of sperm stored by mated females after 24 hours of egg laying. 5B- Co-variance between number of sperm stored in odd-numbered females after 24 hours and the number of offspring produced by these females over 24 hours. Plot created using a loess smooth, to illustrate the non-linear (asymptotic) relationship. Means and 95% C.I. shown.


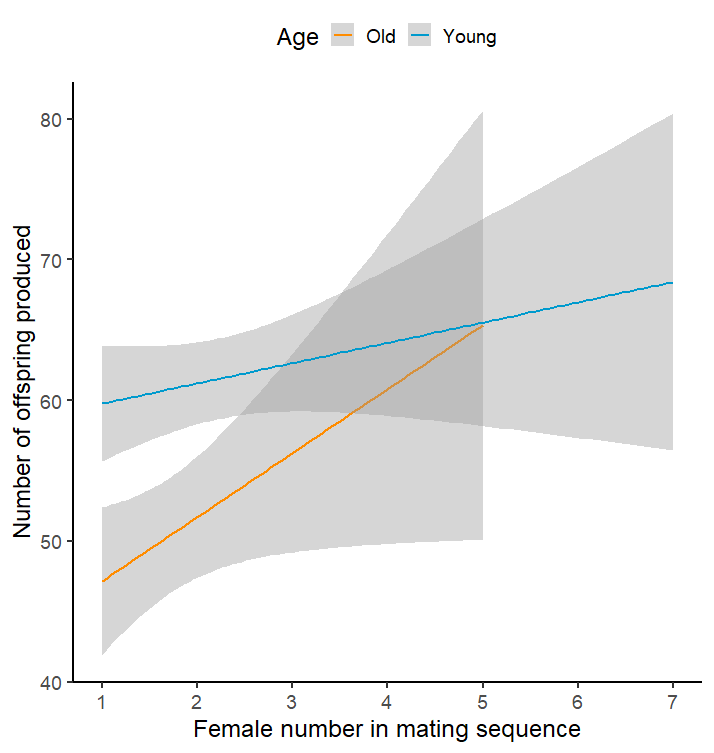


Fig S6: No significant effect of male age or female number in a male’s mating sequence, to affect the number of offspring produced by a female over 24 hours of egg laying, in Experiment C. Females, prior to focal mating with old or young *dah* males, were first mated with *sot* males to provide females with “extra” seminal fluid. Means and 95% C.I. shown.


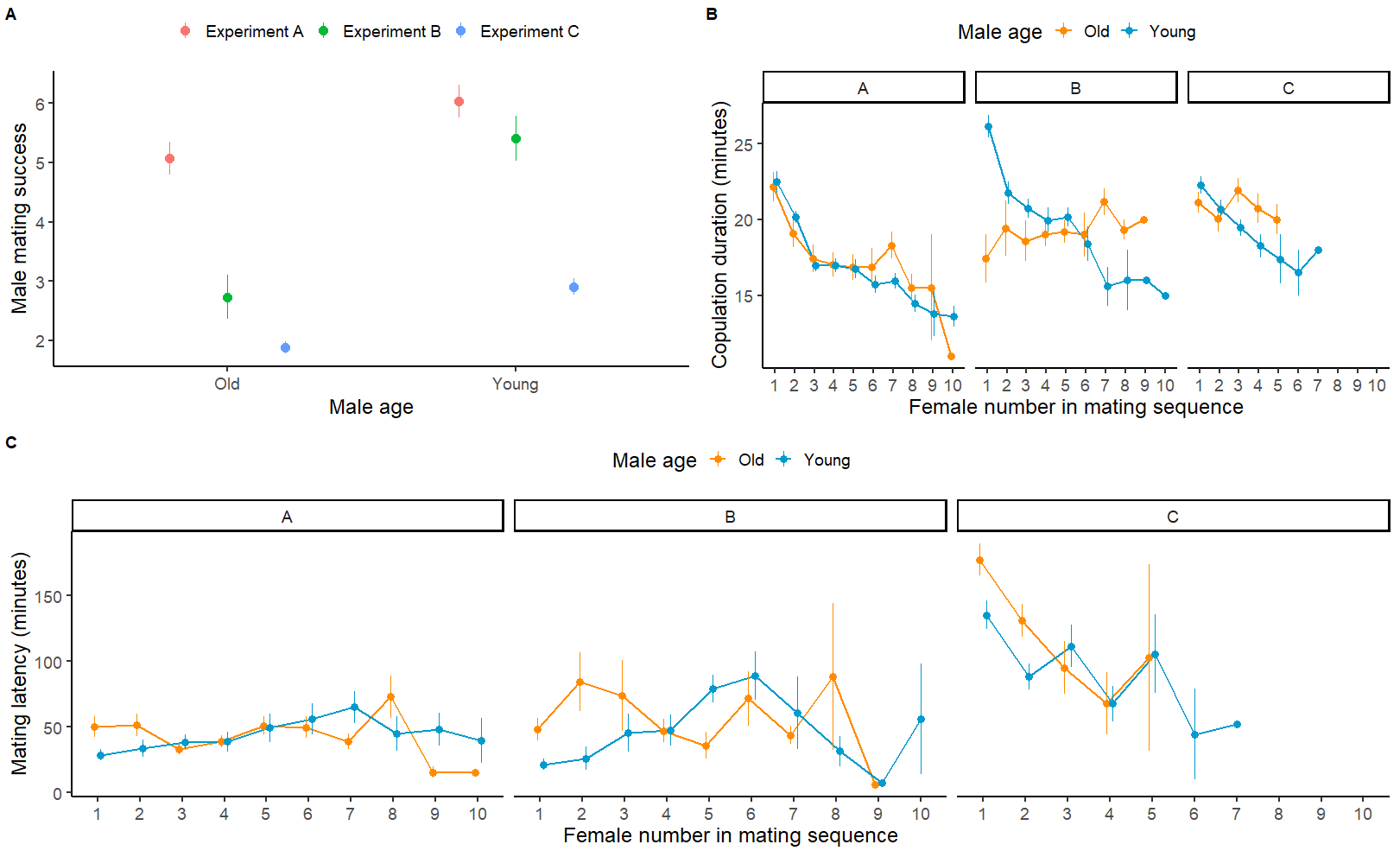


Fig S7: **A**. Mating success of focal old and young males used in our three experiments. **B.** Copulation duration of focal old and young males across Experiments A-C. **C.** Mating latency of focal young and old males across experiments A, B, and C. Means and SEM shown.

Supplementary tables (S1-S9)

Table S1a: sample sizes for number of successful copulations by old and young males with females in a mating sequence, for Experiments A-C. Table S1b: sample sizes for total mating success (i.e. sum of females a male mated with) of old and young males, in Experiments A-C.

| Table 1a | | | | |  | Table 1b | | | | |
| --- | --- | --- | --- | --- | --- | --- | --- | --- | --- | --- |
| Male Age | Female number in mating sequence (i.e. female mating order) | Sample sizes of successful copulations | | |  | Male Age | Male mating success | Sample sizes of males | | |
|  |  | Experiment A | Experiment B | Experiment C |  |  |  | Experiment A | Experiment B | Experiment C |
| Old | Starting male sample size | 60 | 50 | 115 |  | Old | 0 | 3 | 5 | 26 |
| Old | 1^st^ | 57 | 45 | 89 |  | Old | 1 | 5 | 24 | 35 |
| Old | 2^nd^ | 52 | 21 | 54 |  | Old | 2 | 3 | 6 | 35 |
| Old | 3^rd^ | 49 | 15 | 19 |  | Old | 3 | 4 | 1 | 11 |
| Old | 4^th^ | 45 | 14 | 8 |  | Old | 4 | 10 | 5 | 6 |
| Old | 5^th^ | 35 | 9 | 2 |  | Old | 5 | 13 | 0 | 2 |
| Old | 6^th^ | 22 | 9 |  |  | Old | 6 | 7 | 3 |  |
| Old | 7^th^ | 15 | 6 |  |  | Old | 7 | 9 | 3 |  |
| Old | 8^th^ | 6 | 3 |  |  | Old | 8 | 4 | 2 |  |
| Old | 9^th^ | 2 | 1 |  |  | Old | 9 | 1 | 1 |  |
| Old | 10^th^ | 1 |  |  |  | Old | 10 | 1 |  |  |
| Young | Starting male sample size | 60 | 28 | 85 |  | Young | 0 | 0 | 1 | 11 |
| Young | 1^st^ | 60 | 27 | 74 |  | Young | 1 | 0 | 1 | 7 |
| Young | 2^nd^ | 60 | 26 | 67 |  | Young | 2 | 3 | 0 | 22 |
| Young | 3^rd^ | 57 | 26 | 45 |  | Young | 3 | 3 | 4 | 22 |
| Young | 4^th^ | 54 | 22 | 23 |  | Young | 4 | 12 | 3 | 18 |
| Young | 5^th^ | 42 | 19 | 5 |  | Young | 5 | 9 | 4 | 3 |
| Young | 6^th^ | 33 | 15 | 2 |  | Young | 6 | 8 | 10 | 1 |
| Young | 7^th^ | 25 | 5 | 1 |  | Young | 7 | 10 | 3 | 1 |
| Young | 8^th^ | 15 | 2 |  |  | Young | 8 | 6 | 0 | 0 |
| Young | 9^th^ | 9 | 2 |  |  | Young | 9 | 4 | 0 | 0 |
| Young | 10^th^ | 5 | 2 |  |  | Young | 10 | 5 | 2 | 0 |

Table S2: Descriptions of the best-fit models (final) models for each analysis in our study, with details on model aims, dependent and fixed terms, random effects, model error structure, and marginal variance explained. Model outputs of each best-fit model can be found in Tables S3-S9 below.

| Experiment | Aim | Dependent term | Fixed effects | Random effects | Error distribution | $R_{\mathrm{marginal}}^{2}$% |
| --- | --- | --- | --- | --- | --- | --- |
| A | Compare reproductive output of old and young *dah* males in a mating sequence | Number of offspring produced by female | Male age * female number + I(female number^2) + block | 1\|Male ID + 1\|Observation-level | Zero-inflated Poisson | 7.7 |
| B | Compare reproductive output of old and young *gfp* males in a mating sequence | Number of offspring produced by female | Male age + female number + block | 1\|Male ID + 1\|Observation-level | Zero-inflated Poisson | 6.4 |
|  | Compare sperm in SV of old and young *gfp* males with varying mating success | Number of sperm in SV of males | Male age * mating success + I(mating success ^2) + block | 1\|Observation-level | Poisson | 75.7 |
|  | Compare sperm transferred by old and young *gfp* males in a mating sequence | Number of sperm in females frozen within 30 minutes of mating | Male age * female number + block | 1\|Male ID + 1\|Observation-level | Zero-inflated Poisson | 25.7 |
|  | Compare sperm stored by females mated to old and young *gfp* males in a mating sequence | Number of sperm in females frozen 24 hours after mating | Male age * female number + I(female number^2) + block | 1\|Male ID + 1\|Observation-level | Poisson | 21.9 |
|  | Compare accessory gland size of old and young *gfp* males with varying mating success | Accessory gland size | Male age * mating success + block |  | Gaussian | 89.7 |
| C | Compare reproductive output of old and young *dah* males in a mating sequence, when females are first mated to *sot* males | Number of offspring produced by female | Male age + female number + block | 1\|Male ID + 1\|Observation-level | Zero-inflated Poisson | 1 |

Table S3: Effects of male age and female number in a male’s mating sequence, on the number of offspring produced by mated females in Experiment A. Model constructed with zero inflated Poisson error distribution. Two-way interaction model used to interpret interaction only, main-effects model used to interpret main-effects only when two-way interaction is non-significant. Effects of interest highlighted in grey, significant P values of interest in bold.

| Two-way interaction model |  |  |  |  |
| --- | --- | --- | --- | --- |
| Fixed effects | Estimate | SE | z | P |
| (Intercept) | 3.813 | 0.073 | 52.250 | <0.001 |
| I(Female number^2) | -0.014 | 0.004 | -3.430 | 0.001 |
| Age (Young) | 0.318 | 0.075 | 4.240 | <0.001 |
| Female number | 0.109 | 0.033 | 3.300 | 0.001 |
| RepA2 | 0.116 | 0.039 | 2.990 | 0.003 |
| Age (Young)*Female number | -0.043 | 0.019 | -2.240 | **0.025** |
|  |  |  |  |  |
| Random effects | Variance | SD |  |  |
| Male ID | 0.006 | 0.078 |  |  |
| Observation level | 0.146 | 0.382 |  |  |

Table S4: Effects of male age and female number in a male’s mating sequence, on the number of offspring produced by mated females in Experiment B. Model constructed with zero inflated Poisson error distribution. Two-way interaction model used to interpret interaction only, main-effects model used to interpret main-effects only when two-way interaction is non-significant. Effects of interest highlighted in grey, significant P values of interest in bold.

| Two-way interaction model |  |  |  |  |
| --- | --- | --- | --- | --- |
| Fixed effects | Estimate | SE | z | P |
| (Intercept) | 3.923 | 0.111 | 35.260 | <0.001 |
| Age (Young) | 0.266 | 0.126 | 2.110 | 0.035 |
| Female number | -0.037 | 0.030 | -1.240 | 0.216 |
| RepB2 | 0.112 | 0.108 | 1.040 | 0.299 |
| RepB3 | 0.082 | 0.092 | 0.890 | 0.376 |
| Age (Young)*Female number | -0.046 | 0.040 | -1.140 | 0.254 |
|  |  |  |  |  |
| Random effects | Variance | SD |  |  |
| Male ID | 0.000 | 0.000 |  |  |
| Observation level | 0.141 | 0.375 |  |  |
| Main-effects model |  |  |  |  |
| Fixed effects | Estimate | SE | z | P |
| (Intercept) | 3.979 | 0.101 | 39.540 | <0.001 |
| Age (Young) | 0.149 | 0.074 | 2.010 | **0.044** |
| Female number | -0.064 | 0.020 | -3.230 | **0.001** |
| RepB2 | 0.124 | 0.108 | 1.150 | 0.250 |
| RepB3 | 0.083 | 0.093 | 0.900 | 0.370 |

Table S5: Effects of male age and male mating success, on the number of sperm accumulated in seminal vesicles of males in experiment B (one data point per male). Model constructed with Poisson error distribution. Two-way interaction model used to interpret interaction only, main-effects model used to interpret main-effects only when two-way interaction is non-significant. Effects used for interpretation highlighted in grey, significant P values of interest in bold.

| Two-way interaction model |  |  |  |  |
| --- | --- | --- | --- | --- |
| Fixed effects | Estimate | SE | z | P |
| (Intercept) | 9.632 | 0.165 | 58.350 | <0.001 |
| I(Mating success^2) | 0.035 | 0.007 | 4.990 | <0.001 |
| Age (Young) | -1.483 | 0.157 | -9.420 | <0.001 |
| Mating success | -0.377 | 0.062 | -6.120 | <0.001 |
| RepB2 | 0.144 | 0.158 | 0.910 | 0.363 |
| RepB3 | 0.514 | 0.143 | 3.600 | <0.001 |
| Age (Young)*Mating success | -0.166 | 0.036 | -4.630 | **<0.001** |
|  |  |  |  |  |
| Random effects | Variance | SD |  |  |
| Observation level | 0.179 | 0.423 |  |  |

Table S6: Effects of male age and female number in a male’s mating sequence, on the number of sperm transferred by males to mated females in Experiment B. Model constructed with zero inflated Poisson error distribution. Two-way interaction model used to interpret interaction only, main-effects model used to interpret main-effects only when two-way interaction is non-significant. Effects used for interpretation highlighted in grey, significant P values of interest in bold.

| Two-way interaction model |  |  |  |  |
| --- | --- | --- | --- | --- |
| Fixed effects | Estimate | SE | z | P |
| (Intercept) | 4.866 | 0.569 | 8.554 | <0.001 |
| Age (Young) | 2.433 | 0.594 | 4.099 | <0.001 |
| Female number | -0.033 | 0.115 | -0.288 | 0.773 |
| RepB2 | 0.182 | 0.526 | 0.346 | 0.730 |
| RepB3 | 0.675 | 0.461 | 1.464 | 0.143 |
| Age (Young)*Female number | -0.414 | 0.142 | -2.905 | **0.004** |
|  |  |  |  |  |
| Random effects | Variance | SD |  |  |
| Male ID | 0.631 | 0.795 |  |  |
| Observation level | 1.421 | 1.192 |  |  |

Table S7: Effects of male age and female number in a male’s mating sequence, on the number of sperm transferred stored by mated females in Experiment B, after 24 hours of egg laying. Model constructed with Poisson error distribution. Two-way interaction model used to interpret interaction only, main-effects model used to interpret main-effects only when two-way interaction is non-significant. Effects used for interpretation highlighted in grey, significant P values of interest in bold.

| Two-way interaction model |  |  |  |  |
| --- | --- | --- | --- | --- |
| Fixed effects | Estimate | SE | z | P |
| (Intercept) | 3.488 | 0.706 | 4.939 | <0.001 |
| I(Female number^2) | -0.032 | 0.039 | -0.821 | 0.412 |
| Age (Young) | 2.700 | 0.644 | 4.195 | <0.001 |
| Female number | -0.053 | 0.326 | -0.162 | 0.872 |
| RepB2 | -0.304 | 0.651 | -0.467 | 0.641 |
| RepB3 | 0.680 | 0.573 | 1.188 | 0.235 |
| Age (Young)*Female number | -0.373 | 0.184 | -2.030 | **0.042** |
|  |  |  |  |  |
| Random effects | Variance | SD |  |  |
| Male ID | 0.746 | 0.864 |  |  |
| Observation level | 4.244 | 2.060 |  |  |

Table S8: Effects of male age and male mating success, on the area of male accessory glands (cm^2^) in experiment B (one data point per male). Model constructed with Gaussian error distribution. Two-way interaction model used to interpret interaction only. Effects used for interpretation highlighted in grey, significant P of interest values in bold.

| Two-way interaction model |  |  |  |  |
| --- | --- | --- | --- | --- |
| Fixed effects | Estimate | SE | z | P |
| (Intercept) | 0.372 | 0.015 | 24.724 | <0.001 |
| Age (Young) | -0.161 | 0.014 | -11.677 | <0.001 |
| Mating success | -0.040 | 0.003 | -11.831 | <0.001 |
| RepB2 | -0.011 | 0.014 | -0.785 | 0.437 |
| RepB3 | 0.027 | 0.014 | 1.910 | 0.064 |
| Age (Young)*Mating success | 0.027 | 0.004 | 6.577 | **<0.001** |
|  |  |  |  |  |

Table S9: Effects of male age and female number in a male’s mating sequence, on the number of offspring produced by mated females in Experiment C. Model constructed with zero inflated Poisson error distribution. Two-way interaction model used to interpret interaction only, main-effects model used to interpret main-effects only when two-way interaction is non-significant. Effects of interest highlighted in grey, significant P values of interest in bold.

| Two-way interaction model |  |  |  |  |
| --- | --- | --- | --- | --- |
| Fixed effects | Estimate | SE | z | P |
| (Intercept) | 3.953 | 0.059 | 66.540 | <0.001 |
| Age (Young) | 0.154 | 0.074 | 2.080 | 0.038 |
| Female number | 0.055 | 0.029 | 1.940 | 0.053 |
| RepC2 | 0.038 | 0.034 | 1.120 | 0.264 |
| Age (Young)*Female number | -0.050 | 0.034 | -1.460 | 0.145 |
|  |  |  |  |  |
| Random effects | Variance | SD |  |  |
| Male ID | 0.000 | 0.020 |  |  |
| Observation level | 0.083 | 0.288 |  |  |
|  |  |  |  |  |
| Main-effects model |  |  |  |  |
| Fixed effects | Estimate | SE | z | P |
| (Intercept) | 4.015 | 0.041 | 96.820 | <0.001 |
| Age (Young) | 0.059 | 0.036 | 1.650 | 0.099 |
| Female number | 0.020 | 0.016 | 1.300 | 0.193 |
| RepC2 | 0.037 | 0.034 | 1.080 | 0.280 |

Appendix

Appendix 1: Scenarios for ‘male age x female number’ interactions

Some possible scenarios (Appendix figure 1) for how male age could interact with female number in a male’s multiple-mating sequence, to affect male reproductive output. We assume that male reproductive output (*W*) is a negative function of progression through the mating sequence, such that $W\left( i \right)<W(i+1)$ , where *i* is the *i*th female in a mating sequence. For simplicity we assume that the function is linear; i.e. $W=-ax+b$, where x is the order of female in the mating sequence, a is the slope, and b is the intercept. The null hypothesis is that old and young males have the same size and quality of ejaculates, and allocate ejaculates in similar ways to females in their mating sequence, thus produce similar numbers of offspring across a mating sequence:

$$\int_{1}^{10} W_{O}\left( x \right)dx=\int_{1}^{10} W_{Y}\left( x \right)dx$$

Scenario A: Under scenarios of reproductive senescence, the general prediction is that old males have overall lower total reproductive success than young males (i.e. lower intercepts; *b_O_<b_Y_*)). However, old and young males do not differ in their slopes ($a_{O}=a_{Y}$) of decline through a mating sequence and allocate similar proportions of their ejaculate to each female. Here:

$$\int_{1}^{10} W_{O}\left( x \right)dx<\int_{1}^{10} W_{Y}\left( x \right)dx$$

where *W_O_* and *W^Y^* are the reproductive output functions of old and young males over a mating sequence of 10 available females, respectively.

Scenario B: Old males due to fewer mating opportunities in the future, might terminally invest in reproduction, allocating a higher proportion of ejaculate to early than late females compared to young males (i.e. $\left| a_{O} \right|>|a_{Y}|)$. This would lead to relatively higher reproductive output of old males early in a mating sequence, but steeper slopes of decline through the mating sequence, compared to young males.

Scenario C: Old males, due to having lower quantities of ejaculates, might more prudently allocate ejaculates to each female ($i.e.\left| a_{O} \right|<|a_{Y})$. This would lead old males to have lower intercepts but shallower slopes of decline in reproductive output than young males.

Scenario D: Old males have smaller ejaculates than young males, however, they would transfer the same size of ejaculates to females as young males until old males run out of ejaculates. The point where old males run out of ejaculates would be sooner than that of young males, and old males would not have any reproductive output beyond this point.


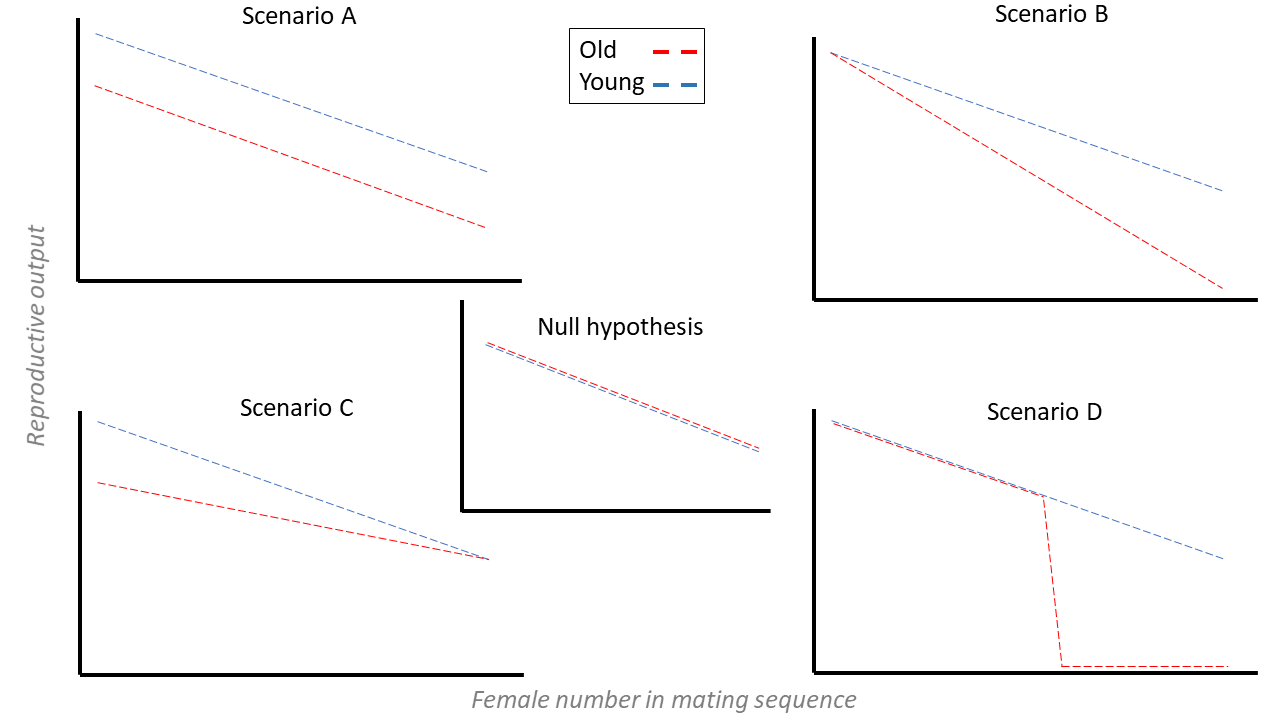


Appendix figure 1: Possible scenarios for how advancing male age can interact with female order in the male’s mating sequence, to influence male reproductive output.

Appendix 2: *son-of-tudor* crossing scheme

*Sot* males used in experiment C were generated by mating brown-eyed straight-winged homozygous *Tudor* females (backcrossed into *dah* background), to *dah* males. To ensure that this crossing scheme produced sterile flies, we used a sub-set of 120 non-experimental virgin *sot* males (3-4 days old), and kept them in bottles containing virgin *dah* females (~20-30 males and females per bottle), for 14 days. None of the bottles had any larvae developing after 14 days, indicating that the males were sterile. We additionally dissected three randomly chosen *sot* males and examined their testes and AGs under a light microscope (Appendix figure 2), revealing the testes and SV to be shrunk and without sperm, but AGs appearing normal, indicating that the males were sterile but producing SF.


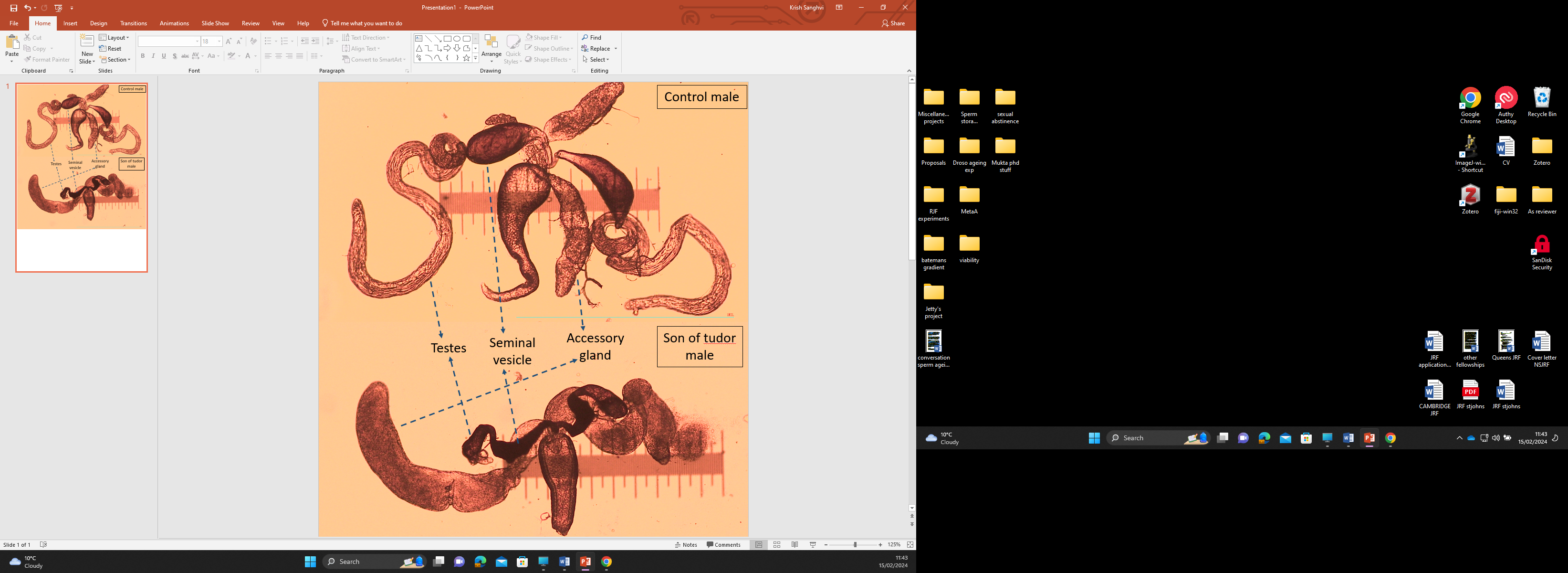


Appendix figure 2: Disrupted testis and seminal vesicles (SV) with no sperm, but normal accessory glands (AG), in *sot* male, compared to control male. Scale = 1mm. Magnification = 40x (4x objective, 10x eyepiece).

Appendix 3: male dissections and imaging

Males in experiment B were dissected, and later imaged to estimate sperm numbers stored in male seminal vesicles, and the size of male AGs. To dissect each male, three droplets of 50uL of PBS were placed on a slide covered with an aqueous solution of gelatine and chromium potassium sulphate (5g gelatine, 0.5g CrK(SO_4_)_2_ dissolved in 1L water at 45°C). The reproductive tract of males (testes, accessory glands, seminal vesicles, and ejaculatory bulb) were separated from the rest of the body, by carefully pulling away the upper abdomen of the male using Inox Biology forceps. The male’s reproductive tract was washed in a second droplet of PBS and surrounding tissue separated. A clean sample of only reproductive tissue was placed on the third droplet. The accessory glands were then separated from the rest of the reproductive tract using microneedles (0.1mm thick), and placed on a new slide which had a measuring scale, inside a droplet of 5uL of PBS. The two accessory glands of each male were immediately imaged without a coverslip on, using the brightfield setting on a Nikon Eclipse 50i microscope with magnification of 4x (objective) and 10x (eyepiece), with each image calibrated to the scale of 1mm. Then, the male’s seminal vesicles were punctured, and sperm present in both seminal vesicles were carefully spread in the droplet of PBS using microneedles. A coverslip was then placed on this sample and then glued to the slide using rubber cement. The sperm were imaged the following day using a 5x air objective on a Zeiss LSM880 confocal laser scanning unit microscope (laser strength = 20; pinhole size = 20.1; Laser wavelength = 488; Pixel strength = 1196 x 1196; objective magnification = 5x, eyepiece magnification = 10x) and Zen black software.

To measure the area of accessory glands on FIJI/ImageJ win32, we used the freehand selection tool to outline the area of each accessory gland separately, and then used the “measure” option under “analyze” to measure the area of each accessory gland. To estimate the number of sperm in the seminal vesicles of males, we used the find maxima plugin (Prominence = 5000, strict setting, output type = single point). The prominence was chosen as 5000 because this point was where the power function relationship between prominence and estimates of sperm number by the find maxima plugin, became linear and flat (Appendix figure 3A, based on visual inspection of 5 samples). To ensure robustness of sperm numbers in male SV estimated by find maxima, we manually counted (using cell counter on FIJI) sperm in eight images of dissected male SV. Repeatability between manual counts and find maxima estimates of sperm numbers in male SV was high (R^2^ = 0.99, Appendix figure 3B).


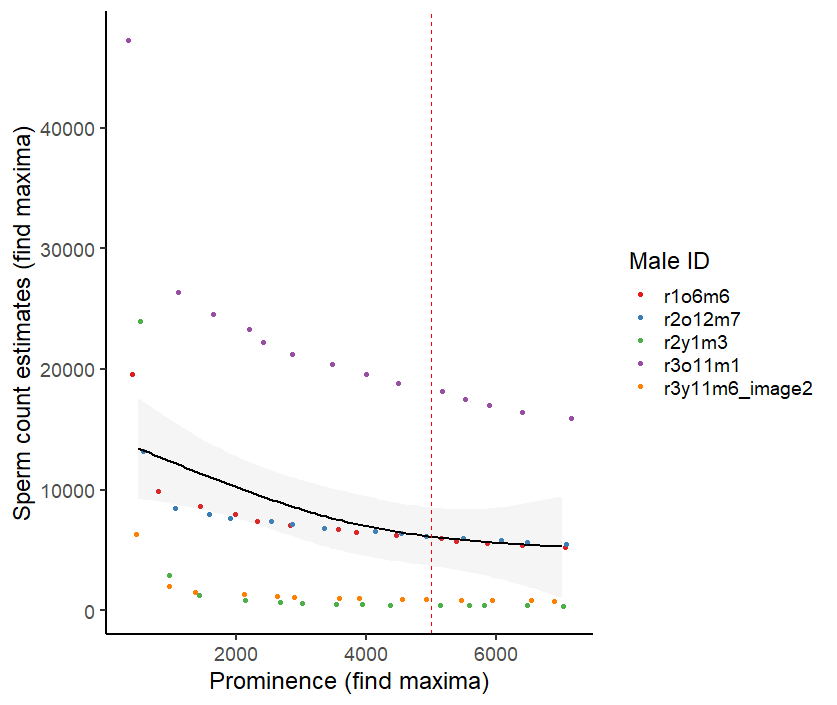


A

B

Appendix figure 3: A. prominence on find maxima chosen as 5000 when estimating sperm stored in males, because this is where the power function relationship between prominence and estimates of sperm number by the find maxima plugin, became linear and flat based on visual inspection. B: High repeatability score of R^2^ = 0.99 between sperm numbers estimated manually (cell counter plugin) versus using find maxima plugin on FIJI.

Appendix 4: Female dissections

Odd-numbered females (frozen after 24 hours) in a *gfp* male’s mating sequence, were dissected to count the number of sperm stored by a female in her long-term sperm storage organs. Even-numbered females in a *gfp* male’s mating sequence (frozen after 30 minutes), were dissected to count the number of sperm transferred by a male to a female.

To dissect frozen females, three droplets of 50uL of Phosphate buffer solution each, were placed on a slide coated with an aqueous solution of gelatine and chromium potassium sulphate. Each female to be dissected was placed on the first droplet, and her reproductive tract was removed gently by separating the last two abdominal segments from the rest of her body, using Inox Biology forceps. Her reproductive organs (bursa, seminal receptacle, and spermathecae) were washed in the second droplet of PBS, and extra surrounding tissue removed. The reproductive organs were then placed on a third droplet. Here, using microneedles (0.1mm thick), the two spermathecae and seminal receptacle were gently spread and the sample covered with a coverslip. Rubber cement (fixogum) was used to glue the coverslip edges to the slide for imaging (Appendix 5) conducted on the following day.

Appendix 5: sperm imaging and counts in females

For odd numbered females (frozen after 24 hours of mating), female reproductive tracts (spermathecae and seminal receptacle) fixed on a slide (described in Appendix 4) were imaged using a Nikon Eclipse50i fluorescence microscope (magnification = 10x objective, 10x eyepiece, wavelength = 480nm) with a chromix HD camera, under UV light from a CoolLED pe300 light source. The number of sperm heads (which appeared as fluorescent green under UV light) were later counted manually from images, using the cell counter plugin on FIJI/ImageJ version win32 (Schindelin et al, 2012). The GFP label in *gfp* flies is expressed at the *Mst35Ba* and *Mst35Bb* loci (Manier et al, 2010). To ensure repeatability of the manual counts, an independent analyst (TLC) counted 30 randomly chosen samples blind to counts made by the first analyst. Repeatability between the counts by the two analysts was high (R^2^ = 0.98, Appendix figure 4A). For even numbered females (frozen within 30 mins of mating), the bursa, spermathecae, and seminal receptacle, were imaged using a 5x air objective on a Zeiss LSM880 confocal laser scanning unit microscope (laser strength = 15; pinhole size = 34.1; laser wavelength = 488nm; pixel strength = 1196 x 1196, objective magnification = 5x, eyepiece magnification = 10x), and the Zen black v14.0.18.201 software. Images were analysed with ImageJ win32, and the find maxima plugin (with prominence = 10000, strict setting, output type = single point) was used to estimate the number of fluorescent green sperm heads in each sample. The prominence was chosen as 10000 because this was the point where the power function relationship between prominence and estimates of sperm number by the find maxima plugin, became linear and flat (Appendix figure 5, based on 8 samples, following Jahn et al (2021)). To ensure that sperm numbers estimated by the find maxima plugin were robust, we tested for repeatability between sperm number counted manually using the cell counter plugin, and sperm number estimated by the find maxima plugin. This repeatability test was done on a subset of 24 randomly chosen samples of even-numbered females (repeatability: R^2^ = 0.98, Appendix figure 4B). Examples of images of dissected individuals described in Appendix 3-5 are provided in Appendix figure 6.

Appendix figure 4: A: high repeatability score of R^2^ = 0.98 between two analysts, when counting sperm numbers stored in odd-numbered females after 24 hours, using the cell counter plugin. B: high repeatability score of R^2^ = 0.98, between sperm numbers when sperm are manually counted using the cell counter plugin, versus estimated using the find maxima plugin, for sperm transferred to even-numbered females.


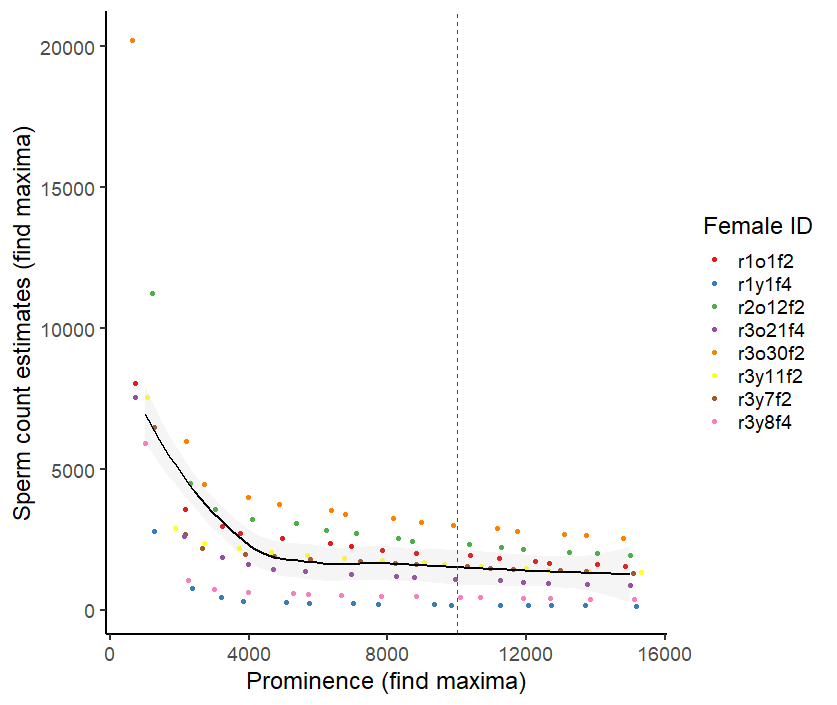


Appendix figure 5: prominence on find maxima chosen as 10000, when estimating sperm stored in females, because this is where the power function relationship between prominence and estimates of sperm number by the find maxima plugin, became linear and flat based on visual inspection.


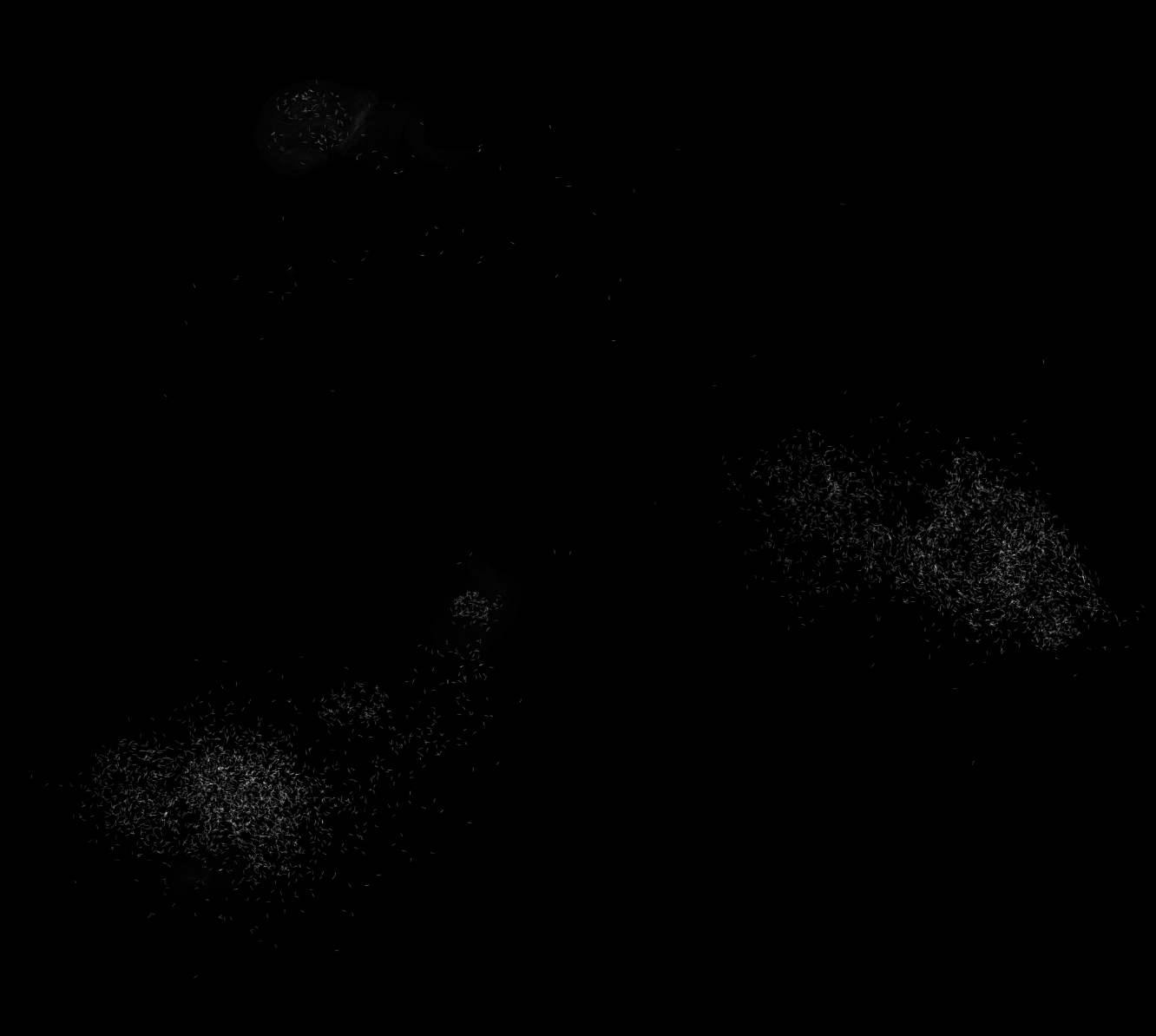

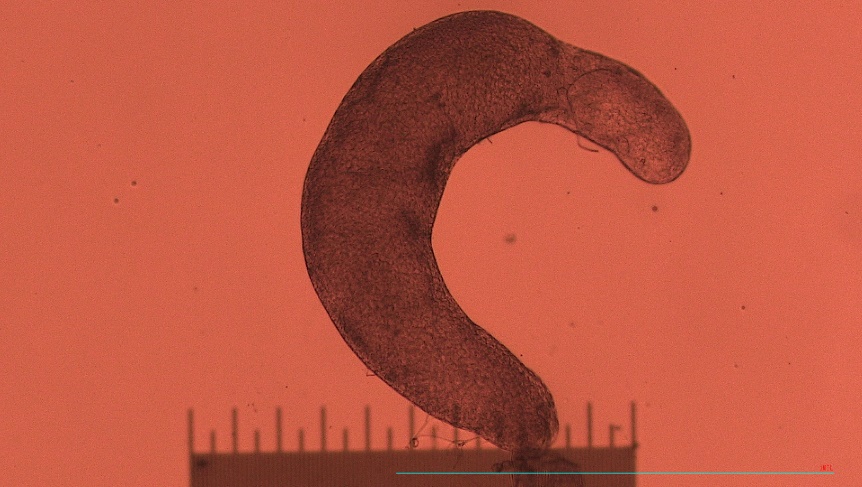

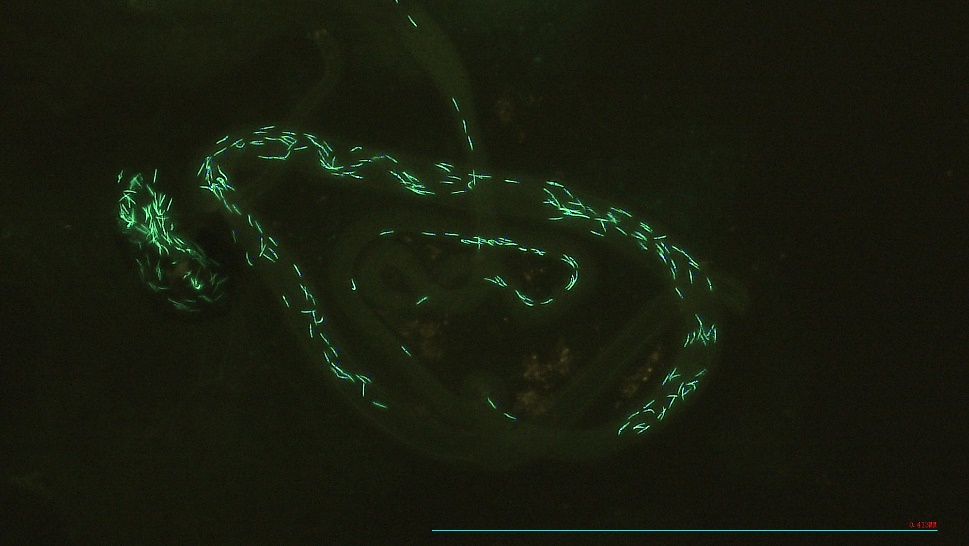

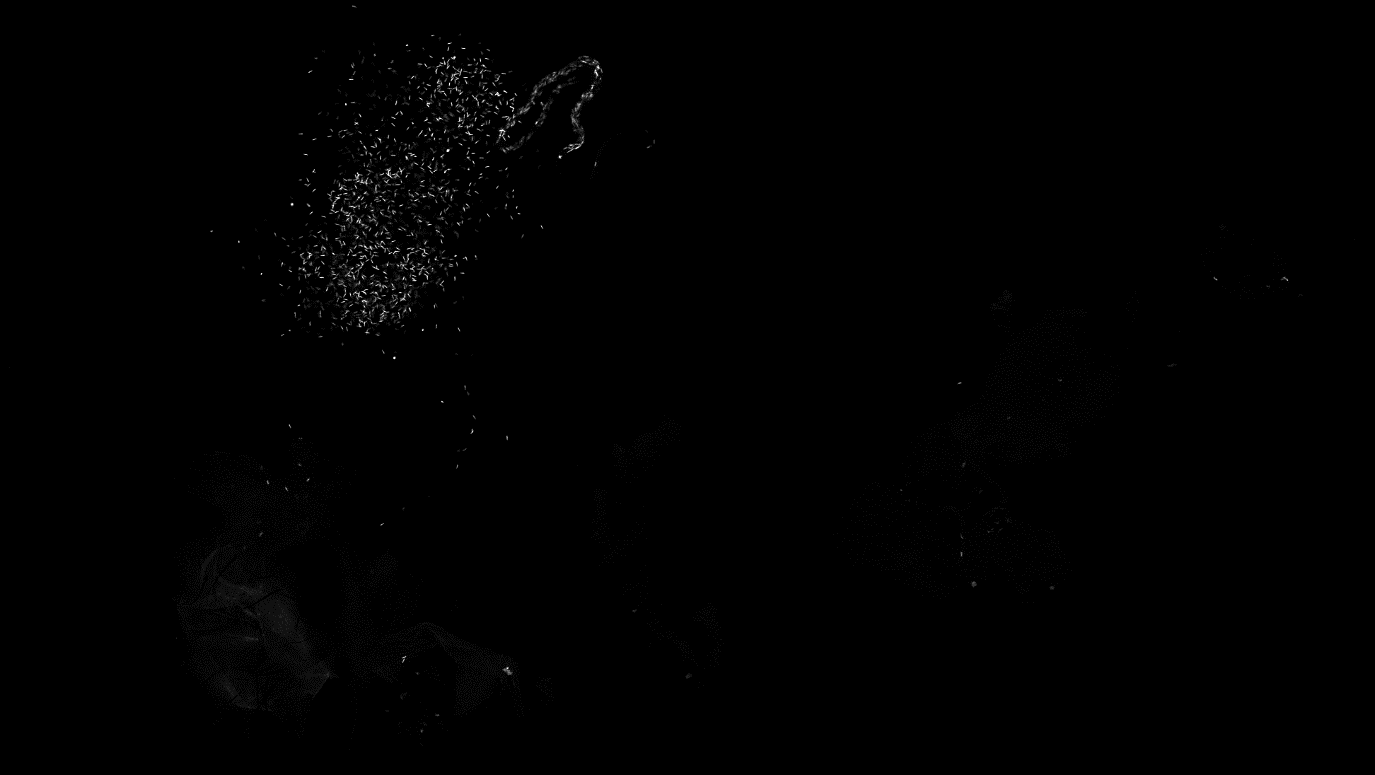


Sperm in spermathecae

Sperm from two seminal vesicles

D

C

B

A

Accessory gland

Sperm in bursa

Sperm in seminal receptacle

Spermathecae

Seminal receptacle

Appendix figure 6: Examples of images from dissected individuals in Experiment B. **A.** sperm in seminal vesicles of males. **B.** accessory gland in male. **C.** sperm in long-term storage organs of even-numbered female after 24 hours. **D.** sperm transferred to odd-numbered female.

Appendix 6: male mating success and latency

We compared the mating success (i.e. total number of females a male mated with) of young and old males in our three experiments. For this, we created a generalised linear model with Poisson error distribution, and included male mating success as our dependent variable. We modelled male age, experiment (A-C), and their two-way interaction as fixed effects, and observation-level ID as a random effect to account for overdispersion. We also compared male mating latency (time elapsed between male being paired with a female, and the start of copulation). For this, we modelled male mating latency as our dependent variable using an LMM with Gaussian error distribution. Male age, a linear and quadratic term of female number, their two-way interactions with male age, and experiment number, were included as fixed effects, with male ID as a random effect.

Old males consistently mated with fewer females compared to young males (z = 6.423, P < 0.001, Figure S7A). However, this difference was greater in Experiments B (age*experiment C: z = 3.491, P < 0.001) and C (age*experiment C: z = 2.027, P < 0.043) than Experiment A. Old males had a lower mating latency than young males early in the mating sequence but not later (age*female number: t = 3.159, P = 0.002, Figure S7B, S7C).

Ejaculate limitation seems to be an unlikely explanation for the lower mating success of old males, because they had more sperm in their SV and larger AGs than young males. Instead, the lower mating success of old males might be a consequence of their longer mating latencies. These longer latencies could be due to old males being less attractive to females or worse at courtship, thus females taking longer to accept mating with an old than a young male (e.g. Amin et al, 2012; Rezaei et al, 2015). Furthermore, differences between old and young males in their mating latencies were greater in Experiment C (when females were first mated to *sot* males) than Experiment B (Figure S7C). This result suggests that females show stronger choice for young males than old males, when females have previously been mated compared to when females are virgins. Comparing male mating success and latency were not a part of our main aims; therefore, we have included these analyses in the appendix only.

Appendix 7: Bateman gradients

Note: These results are only presented as exploratory analyses to generate new hypotheses regarding sexual selection and as such are not a part of our study aims.

We first investigated whether the age of males and seminal fluid availability to females, might influence the strength and slope of the Bateman gradient, to explore whether the strength for pre-copulatory sexual selection is influenced by age and ejaculate limitation. For this, we calculated Bateman gradients, i.e. the slope of the linear relationship between male mating success and reproductive success (Anthes et al, 2017), for old and young males used in Experiments A and C. We first calculated the total mating success (sum of successful copulations by a male in his mating sequence- one data point per male), and total reproductive success (sum of offspring produced by all females that copulated with a male- one data point per male), for each male that mated in Experiments A and C. We then calculated the slopes of the linear regression between male mating success and reproductive success (Appendix figure 7A). Visual inspection indicated that males in Experiment C had steeper slopes than males in Experiment A, suggesting that the opportunity for sexual selection might be greater when females are not seminal fluid limited. Additionally, young males had steeper gradients, suggesting a greater opportunity for pre-copulatory sexual selection in young compared to old males.

Next, we tested for covariances between male reproductive output (i.e. number of offspring produced with each female- multiple data points per male) and total male mating success (i.e. total number of females a male mated with in the mating sequence- one data point per male), as these can lead to a biased estimation of Bateman gradients (Anthes et al, 2017). For example, if males that produce more offspring with each female also mate with more females (for instance, due to being more attractive), then females later in a mating sequence will be exposed to a biased group of high reproductive quality males (i.e. more fertile males). These covariances can inflate or deflate the Bateman gradient. We tested this hypothesis using data from experiment A using a GLMM with zero-inflated Poisson error distribution. We included the number of offspring a male produced with each female in the mating sequence as our dependent variable. We modelled female number in the sequence and its interaction with male total mating success as fixed effects. We additionally included male age and block as fixed effects, and male ID as a random effect. Males who mated with more females also experienced steeper declines in their reproductive output through the mating sequence (female number * total mating success: z = -8.47, P < 0.001; Appendix figure 7B). However, males who mated with more females also produced more offspring early in the mating sequence, compared to males who mated with fewer females (total mating success: z = 3.52, P < 0.002; Appendix figure 7B). Our results reveal positive covariances between initial reproductive output in a mating sequence and male mating success, but negative covariances between initial reproductive output/mating success and later reproductive output. Such covariances between male reproductive output, male mating success, and fertility earlier versus later a mating sequence, can lead to incorrect interpretations of the strength of sexual selection.


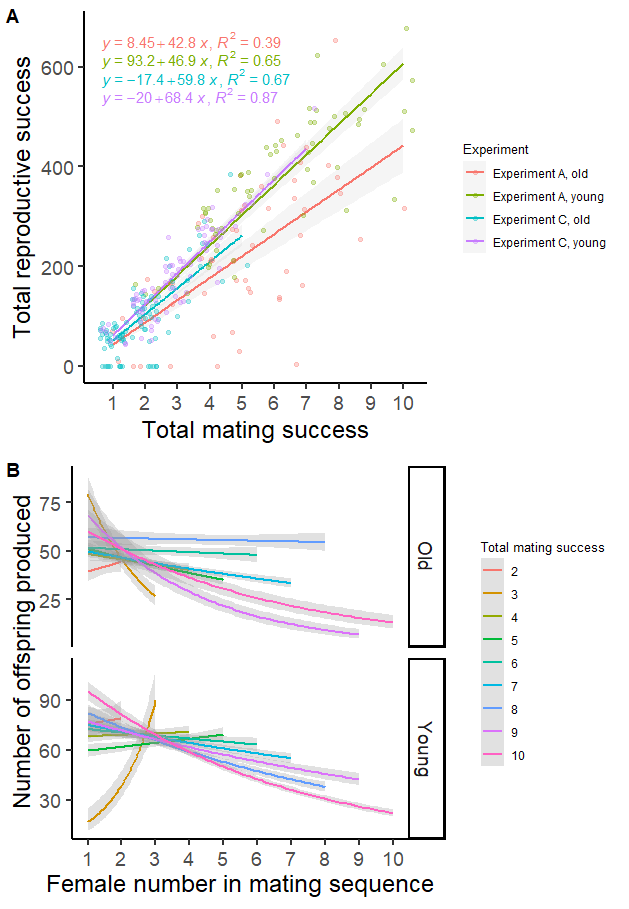


Appendix figure 7: **A.** Bateman’s gradients showing the linear relationship between male mating success and reproductive success, for old and young males used in Experiments A and C. Slopes and R^2^ values shown. Intercepts set to zero to allow direct comparisons of slopes. Each dot represents one male. **B.** Positive covariances between initial reproductive output in a mating sequence and male’s total mating success, but negative covariances between initial reproductive output and later reproductive output in the sequence, using data from experiment A. Dark lines show means, shaded regions show 95% C.I.

**References:**

Anthes, N., Häderer, I. K., Michiels, N. K., & Janicke, T. (2017). Measuring and interpreting sexual selection metrics: Evaluation and guidelines. *Methods in Ecology and Evolution*, *8*(8), 918–931. https://doi.org/10.1111/2041-210x.12707

Jahn, M. T., Lachnit, T., Markert, S. M., Stigloher, C., Pita, L., Ribes, M., Dutilh, B. E., & Hentschel, U. (2021). Lifestyle of sponge symbiont phages by host prediction and correlative microscopy. *The ISME Journal*, *15*(7), 2001–2011. https://doi.org/10.1038/s41396-021-00900-6
